# Patients With Type 2 Diabetes Mellitus and Heart Failure Benefit More From Sodium-Glucose Cotransporter 2 Inhibitor: A Systematic Review and Meta-Analysis

**DOI:** 10.3389/fendo.2021.664533

**Published:** 2021-10-25

**Authors:** Chengcong Chen, Hong Peng, Mingzhu Li, Xiyan Lu, Miao Huang, Yongmei Zeng, Guoqing Dong

**Affiliations:** ^1^ Section of Endocrinology, Department of Pediatrics, Shenzhen Maternity and Child Healthcare Hospital, Shenzhen, China; ^2^ Department of infectious disease, Shenzhen People’s Hospital, Shenzhen, China; ^3^ Section of Gastroenterology, Department of Pediatrics, Shenzhen Maternity and Child Healthcare Hospital, Shenzhen, China

**Keywords:** sodium-glucose cotransporter 2 inhibitor, type 2 diabetes mellitus, heart failure, risk of mortality, hospitalization for heart failure

## Abstract

**Background:**

Patients with type 2 diabetes mellitus (T2DM) and heart failure (HF) are at higher risk of mortality and hospitalization for heart failure (HHF). A recent study showed that sodium-glucose cotransporter 2 (SGLT-2) inhibitors may be a promising choice.

**Methods:**

We searched the PubMed, Embase, and Cochrane databases of clinical trials for randomized controlled trials investigating the long-term effects of SGLT-2 inhibitors in patients with T2DM and HF compared with placebo. The primary outcome was cardiovascular death or HHF, and the secondary outcomes included cardiovascular death (CV death), HHF, and all-cause mortality. We also conducted an exploratory analysis and tried to identify the population, which will benefit more from the treatment.

**Results:**

After the study selection, a total of 5 trials, including 4 subgroup analyses, met the eligibility criteria. The results suggested that the use of SGLT-2 inhibitors was associated with a reduction in the incidence of CV death or HHF (HR, 0.69[95%CI, 0.63-0.77], P<0.00001), CV death (HR, 0.80[95%CI, 0.69-0.92], P = 0.001), HHF (HR, 0.67[95%CI, 0.60-0.76], P < 0.00001), and all-cause mortality (HR, 0.74[95%CI, 0.64-0.86], P < 0.0001). Moreover, patients with T2DM and HF may benefit more from the treatment than those with T2DM/HF.

**Conclusion:**

The long-term use of SGLT-2 inhibitors can help reduce the risk of mortality and HHF in patients with T2DM and HF.

**Systematic Review Registration:**

PROSPERO [https://www.crd.york.ac.uk/prospero/display_record.php?ID=CRD42021233156], identifier [CRD42021233156].

## Introduction

Between 2000 and 2016, there was a 5% increase in premature mortality due to diabetes, and the global prevalence of diabetes rose from 4.7% in 1980 to 8.5% in 2014. In particular, the majority of patients were diagnosed with type 2 diabetes mellitus (T2DM) (1). Heart failure (HF) is another global health issue, and approximately 50% of patients die 5 years after they are diagnosed (2). T2DM is known to represent a risk factor for HF, however, HF is also a risk factor for T2DM (3). People with T2DM are at least twice the risk of HF (4), and HF patients with diabetes are at higher risk of hospitalization and mortality (5). Current treatment strategies usually manage them independently, as metformin is the preferred initial pharmacologic agent for the treatment of T2DM (6), while angiotensin converting enzyme inhibitors, Angiotensin Receptor Blockers and beta blockers are the most common prescription for HF patients (2). However, some studies reported that the sodium-glucose cotransporter 2 (SGLT-2) inhibitor, initially developed as an anti-diabetes drug, might be a promising strategy for the management of HF (7–14).

Recently, the Empire HF trial investigated a 12-week treatment with empagliflozin in HF patients. Although the left ventricular ejection fraction (LVEF) remained unchanged, there were modest reductions in left ventricular and left atrial volumes (15). However, in another trial using empagliflozin for 12 weeks, the N-terminal pro-b-type natriuretic peptide (NT-proBNP), as well as daily activity level or health status were not improved (16).

In the EMPA-RESPONSE-AHF trial, the investigators recruited HF patients with or without diabetes. The participants who received empagliflozin showed a reduction in the combined incidence of worsening HF, rehospitalization for HF, or death at 60 days; however, they failed to reach these end points separately, as well as diuretic response, NT‐proBNP, and some other parameters (11). There are also many trials reported effects of different SGLT-2 inhibitors in HF patients, like canagliflozin (17), Dapagliflozin (9), sotagliflozin (10), luseogliflozin (12), while the others like ertugliflozin (18), ipragliflozin (19) and tofogliflozin (20) are mainly focusing on T2DM patients.

Interestingly, in those studies that enrolled participants with both T2DM and HF, short-term treatment with different SGLT-2 inhibitors did not show an improvement of NT‐proBNP (12, 21, 22). In the SUGAR-DM-HF trial, treatment with empagliflozin for 36 weeks significantly reduced the level of NT-proBNP (13). In addition, a 12-month treatment strategy with canagliflozin also led to improvement of atrial natriuretic peptide (ANP) and brain natriuretic peptide (BNP) (23). In particular, all the trials mentioned above showed that SGLT-2 inhibitors were well tolerated in patients.

These studies demonstrated a trend that longer duration of treatment might help achieve beneficial effects. On the other hand, no trend was obtained concerning the population, which would benefit more from the treatment. Some multicenter, randomized controlled trials reported the long-term effects of SGLT-2 inhibitors in T2DM/HF with or without HF/T2DM. Since there was no systematic review that summarized the long-term incidence of mortality and hospitalization for heart failure (HHF) in patients with T2DM and HF who used SGLT-2 inhibitor, we tried to conduct a systematic review and meta-analysis and identify the population, which would benefit more from the treatment.

## Methods

This systematic review and meta-analysis was conducted according to the Preferred Reporting Items for Systematic Reviews and Meta-Analyses (PRISMA) statement (24, 25) and was registered in PROSPERO, ID CRD42021233156.

### Literature Search and Eligibility Criteria

An electronic search in PubMed, Embase, and Cochrane database of clinical trials was performed to identify randomized controlled trials (RCTs) investigating the use of SGLT-2 inhibitors in patients with T2DM and HF. The search strategy had no language restrictions and was carried out on January 21, 2021. We also performed a manual search of references of systematic reviews and meta-analyses or review articles to identify potential eligible trials. The details of the search strategy are provided in **Supplementary File 1**.

Clinical trials meeting the following criteria were considered as eligible trials: 1) RCTs, including patients aged ≥ 18 years or over who had T2DM and HF; 2) the intervention compared SGLT-2 inhibitors versus placebo regardless of background medication and comorbidities; and 3) the outcomes included the numbers of CV death or HHF. Trials were excluded if they: 1) were randomized crossover trials; 2) had no full text; and 3) were on going trials.

### Study Selection and Data Extraction

After study selection, the following data were collected using a data collection form that had been prepared previously, which included: 1) study design, years of publication, intervention setting, and duration of treatment, 2) patient characteristics, including sample size, age, sex ratio, subtype of HF, and HF status; and 3) outcomes of interest. These processes were performed independently by two investigators (CCC, PH). When discrepancies occurred, consultation with a third investigator (DGQ) was facilitated to reach a consensus.

### Quality Assessment

We used the Cochrane Collaboration’s tool to assess the quality of eligible trials by two independent reviewers (CCC, PH). Risk of bias concerning the following aspects was assessed: random sequence generation, allocation concealment, blinding of participants and personnel, blinding of outcome assessment, incomplete outcome data, selective reporting, and other biases. Supporting views of each bias were drawn from the manuscripts and protocols, if available.

### Data Analysis

We used Review Manager 5.4.1 (Cochrane Library) to conduct the meta-analysis and pooled the hazard ratio (HR) of each outcome into analysis using the generic inverse-variance method. The I^2^ test was used to evaluate heterogeneity, and I^2^ ≥ 50% was defined as significant heterogeneity. Then, the random-effects model was applied if I^2^ ≥ 50%; otherwise, the fixed-effect model was used. P values ≤ 0.05 was considered statistically significant.

The primary outcome was CV death or HHF, and secondary outcomes included CV death, HHF, and all-cause mortality. We also conducted an exploratory analysis to identify which population will benefit more from the treatment by pooling the data from participants with T2DM/HF alone. Safety outcomes were also collected in a summary table.

### Sensitivity Analysis

In order to confirm the results, a range of sensitivity analyses were conducted: 1) in the DECLARE-TIMI 58 trial, patients were identified as having existing HF, with demonstrated HF history but no existing reduced EF. Thus, we conducted sensitivity analyses using data from existing HF participants, combining data of two kinds of participants. 2) An analysis of HHF, in DAPA-HF and EMPEROR-Reduced trials, they reported the outcomes of first and recurrent HHF, and time to first HHF only. 3) Analysis of different subtype of HF. 4) Analysis of different HF status.

## Result

**Figure 1:**
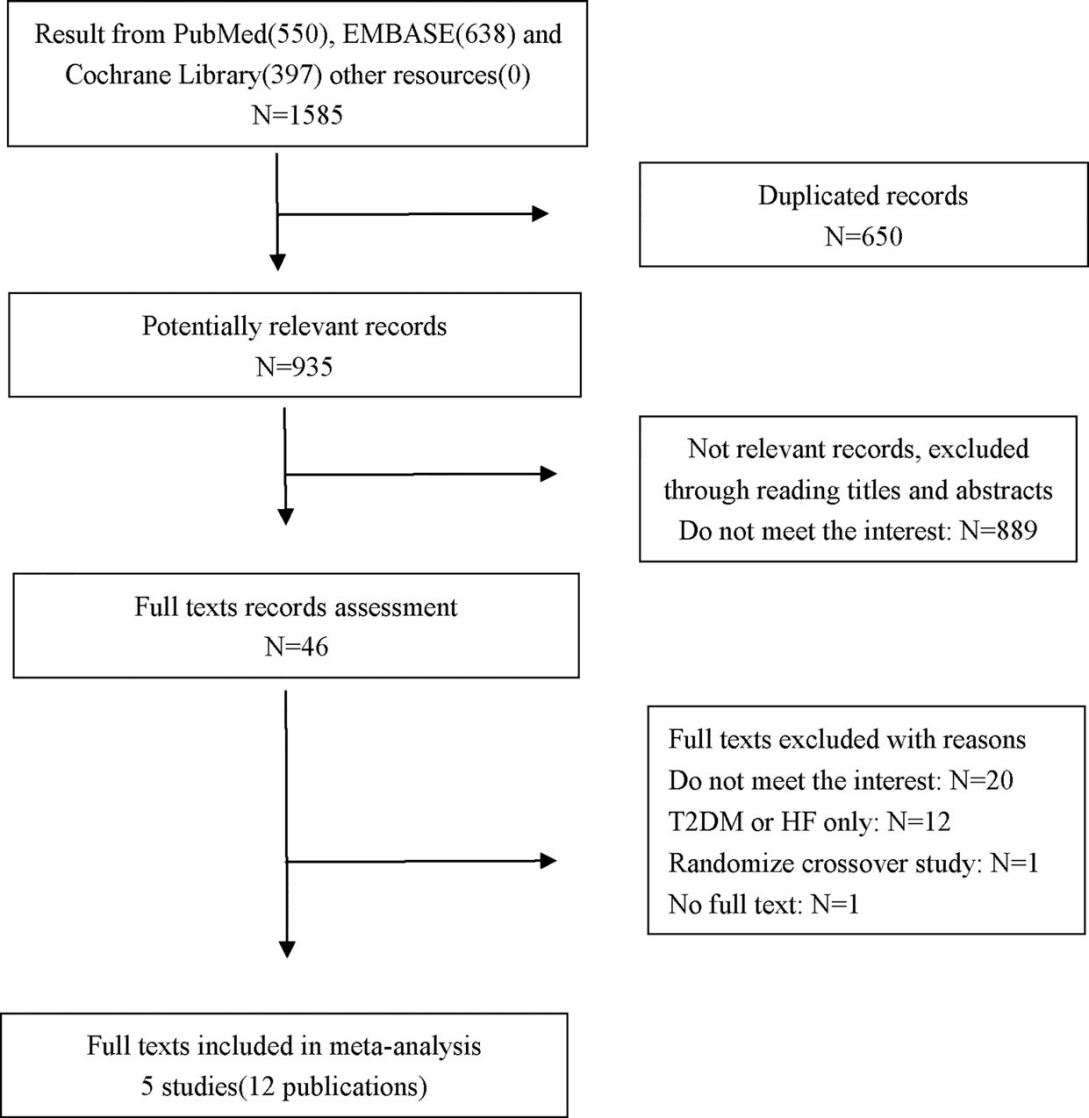
Process of study selection.

A total of 1585 records were identified. After screening, five multicenter double-blind trials (12 publications), including both main studies and their subgroup analyses, met the eligibility criteria (10, 17, 26–28). Among these five trials, four trials recruited patients with T2DM/HF with or without HF/T2DM; thus, the data from their subgroup analyses comparing SGLT-2 inhibitors with placebo in patients with both T2DM and HF were pooled into the meta-analysis. The selection process is shown in **Figure 1**.

### Study Characteristics

Details of the characteristics of the 5 included trials are presented in **Table 1**. Their main studies were published from 2017 to 2020, and all of them were double-blind trials, with multicenter design, and the sample sizes were between 1222 and 17160, with the longest follow-up period being 4.2 years. In the SOLOIST-WHF trial, patients with both T2DM and HF were recruited and treatment with sotagliflozin was investigated (10). The DAPA-HF trial and EMPEROR-Reduced trial recruited HF patients with or without T2DM, and treatment with dapagliflozin and empagliflozin was investigated, respectively (8, 9). In the CANVAS trial, canagliflozin was investigated in T2DM patients with or without HF (7). In the DECLARE-TIMI 58 study, dapagliflozin was investigated in T2DM patients with established atherosclerotic cardiovascular disease (ACD) or had multiple risk factors for ACD (14).

**Table 1:** Study characteristics of the main study of 5 RCTs.

Study	Years	Duration	Population	Intervention	Sample size, n	Age*, years	Female sex, %
CANVAS	2017	Mean 188.2w	T2DM with or without HF	Canagliflozin	5795	63.2 ± 8.3	35.1
				Placebo	4347	63.4 ± 8.2	36.7
DAPA-HF	2019	Media 18.2m	HF with or without T2DM	Dapagliflozin	2373	66.2 ± 11.0	23.8
				Placebo	2371	66.5 ± 19.8	23.0
DECLARE–TIMI 58	2018	Media 4.2y	T2DM with established or multiple risk factors for ACD	Dapagliflozin	8582	63.9 ± 6.8	36.9
				Placebo	8578	64.0 ± 6.8	37.9
EMPEROR-Reduced	2020	Media 16m	HF with or without T2DM	Empagliflozin	1863	67.2 ± 10.8	23.5
				Placebo	1867	66.5 ± 11.2	24.4
SOLOIST-WHF	2020	Media 9m	T2DM with HF	Sotagliflozin	608	69 (63-76)	32.6
				Placebo	614	70 (64-76)	34.9

T2DM, type 2 diabetes mellitus; HF, heart failure; ACD, atherosclerotic cardiovascular disease.

*Age was presented as mean ± SD or median (interquartile range).

In this study, we only included patients with T2DM and HF. Thus, we extracted the data from the subgroup analyses of four trials except the SOLOIST-WHF trial (10, 17, 26–28). The baseline characteristics of these subgroup analyses/main studies are presented in **Table 2**. In the DAPA-HF, DECLARE-TIMI 58, and EMPEROR-Reduced trials, only HF patients with reduced ejection fraction (EF) were included (26–28), while in the CANVAS and SOLOIST-WHF trials, patients with HF were included, regardless of the patients having preserved or reduced EF (10, 17). In particular, in the DAPA-HF and EMPEROR-Reduced trials, participants with existing HF were recruited (26, 27). In the DECLARE–TIMI 58 trial, participants were divided into groups: with existing HF, with history of HF but have no existing reduced EF (28). In the CANVAS and SOLOIST-WHF trials, all patients had a history of HF, but it was not known if they had existing HF during the randomization period (10, 17).

**Table 2:** Study characteristics of the subgroup analyses/main study comparing SGLT-2 inhibitors *VS* placebo in patients with T2DM and HF.

Study	Intervention	Sample size, n	Age, years#	Female sex, %	Type of HF included	HF status
CANVAS	Canagliflozin	803	64.1 ± 8.3	43.1	Both preserved and reduced	With history of HF, but not known if had existing HF.
	Placebo	658	63.4 ± 8.3	45.9		
DAPA-HF	Dapagliflozin	1075	66.3 ± 9.9	22.3	Reduced only	Existing HF
	Placebo	1064	66.7 ± 9.8	22.3		
DECLARE–TIMI 58	Dapagliflozin	318/908*	NR	NR	Reduced only	Including existing HF, with history of HF but without existing reduced EF
	Placebo	353/1007	NR	NR		
EMPEROR-Reduced	Empagliflozin	927	66.8 ± 10.0	22.7	Reduced only	Existing HF
	Placebo	929	66.6 ± 10.3	23.5		
SOLOIST-WHF	Sotagliflozin	608	69 (63-76)	32.6	Both preserved and reduced	Diagnosis with HF ≥3 months prior to Screening, and with history of hospitalization for HF
	Placebo	614	70 (64-76)	34.9		

T2DM, type 2 diabetes mellitus; HF, heart failure; NR, not reported; EF, ejection fraction.

*Existing HF only/Combined existing HF and with history of HF.

^#^Age was presented as mean ± SD or median (interquartile range).

The risk of bias assessment is presented in **Supplementary File 2**. The reasons for each evaluation were drawn from the full texts and/or their protocols. After evaluation, the risk of bias was considered to be low. We did not assess publication bias because there were only five eligible trials.

The outcomes of the subgroup analyses/main study comparing SGLT-2 inhibitors *vs*. placebo in patients with T2DM and HF were collected and summarized in **Table 3**.

**Table 3:** Outcomes of the subgroup analyses/main study comparing SGLT-2 inhibitors *VS* placebo in patients with T2DM and HF.

Study	Intervention	CV death or HHF, n	CV death, n	HHF, n	All caused mortality, n
CANVAS│	Canagliflozin	35.4	24.3	14.1	29.2
	Placebo	56.8	31.6	28.1	38.7
DAPA-HF	Dapagliflozin	213/328*	121	138	143
	Placebo	268/415*	148	172	178
DECLARE–TIMI 58	Dapagliflozin	59/151^#^	25/79^#^	41/92^#^	38/122^#^
	Placebo	95/194^#^	47/85^#^	63/130^#^	68/149^#^
EMPEROR-Reduced	Empagliflozin	200	104	140/221^	NR
	Placebo	265	113	201/337^	NR
SOLOIST-WHF	Sotagliflozin	245	51	194	65
	Placebo	355	58	297	76

T2DM, type 2 diabetes mellitus; HF, heart failure; NR, not reported.

*CV death or HHF/CV death or first and recurrent HHF.

^#^Existing HF only/Combined existing HF and with history of HF.

^Time to first HHF/first and recurrent HHF.

│Data were presented with the unit Patients per 1000 patients-years.

### Outcome–Cardiovascular Death or Hospitalization for Heart Failure

**Figure 2:**
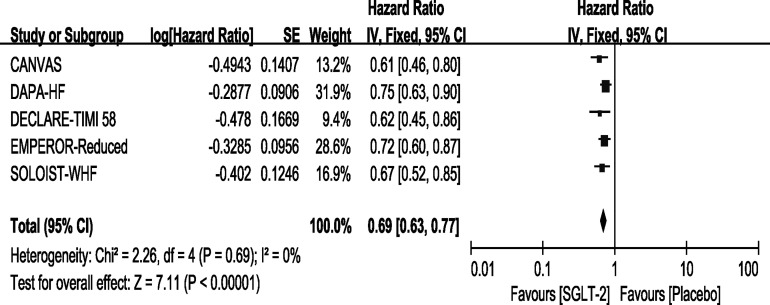
Outcome of CV death or HHF in patients with T2DM and HF.

All the trials included in the study reported that the use of SGLT-2 inhibitors significantly improved the outcome of CV death or HHF, and the result of our meta-analysis consisted with these studies (HR, 0.69[95%CI, 0.63-0.77], P<0.00001) with no heterogeneity (I^2^ = 0, **Figure 2**). In the SOLOIST-WHF trial, the primary outcome was CV death and HHF alongside urgent visits for HF. We pooled this data for analysis because events concerning urgent visits for HF were not reported, and we could not extract the events of CV death and HHF. As described above, we conducted a sensitivity analysis as shown in **Supplementary File 3**, which confirmed our results.

### Outcome—Cardiovascular Death

**Figure 3:**
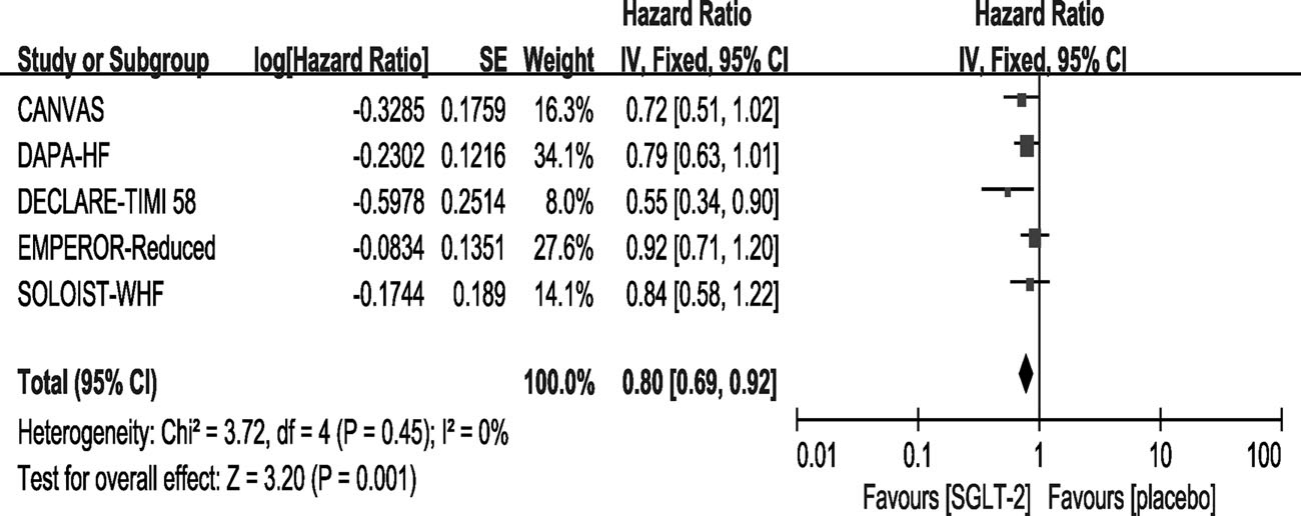
Outcome of CV death in patients with T2DM and HF.

All of the included trials reported events of CV death. However, only the DECLARE-TIMI 58 trial reported a statistically significant improvement concerning CV deaths in the SGLT-2 inhibitor group. In our meta-analysis, the overall effects of the five trials showed that SGLT-2 inhibitors can significantly reduce the rate of CV death [HR, 0.80(95%CI, 0.69-0.92), P = 0.001] with no heterogeneity (I^2^ = 0, **Figure 3**). The sensitivity analysis did not exhibit inconsistency with our results, as shown in **Supplementary File 3**.

### Outcome—Hospitalization for Heart Failure

**Figure 4:**
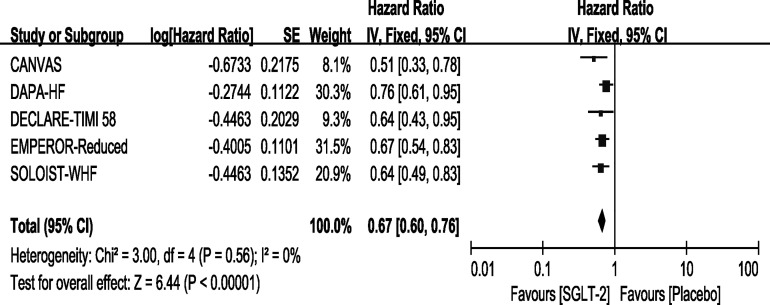
Outcome of HHF in patients with T2DM and HF.

All of the included trials reported the outcome of HHF. Our results showed that use of SGLT-2 inhibitors can reduce the incidence of HHF [HR, 0.67(95%CI, 0.60-0.76), P < 0.00001] with no heterogeneity (I^2^ = 0, **Figure 4**). This result was consistent with the original reports of the five trials. The sensitivity analyses, as shown in **Supplementary File 3**, conformed with the stability of our result.

### Outcome—All Caused Mortality

**Figure 5:**
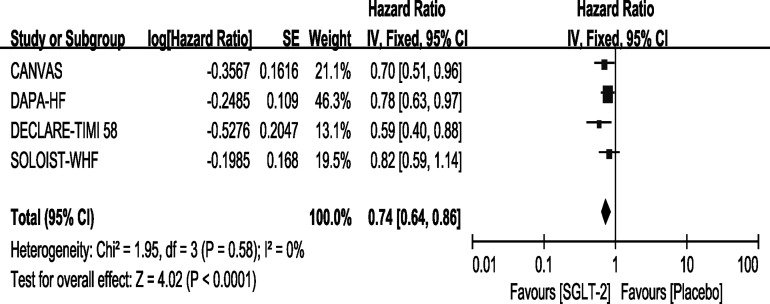
Outcome of all-cause mortality in patients with T2DM and HF.

Except for the EMPEROR-Reduced trial, the remaining four trials reported the outcome of all-cause mortality. Among them, only the SOLOIST-WHF trial showed that Sotagliflozin did not improve the outcome. In our meta-analysis, the use of SGLT-2 inhibitors can reduce the rate of all-cause mortality compared with placebo [HR, 0.74(95%CI, 0.64-0.86), P < 0.0001], with no heterogeneity (I^2^ = 0, **Figure 5**). The sensitivity analyses, as shown in **Supplementary File 3**, conformed with the stability of our result.

### Outcome—Exploratory Analysis in Participants With T2DM/HF Alone

**Figure 6:**
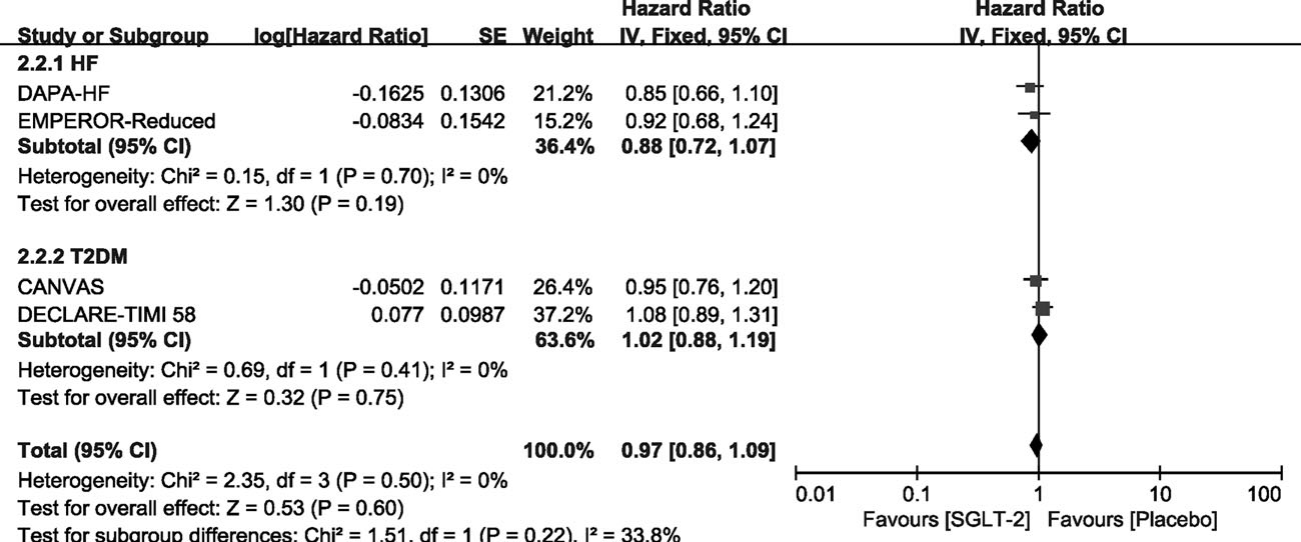
Outcome of CV death in patients with T2DM or HF.

**Figure 7:**
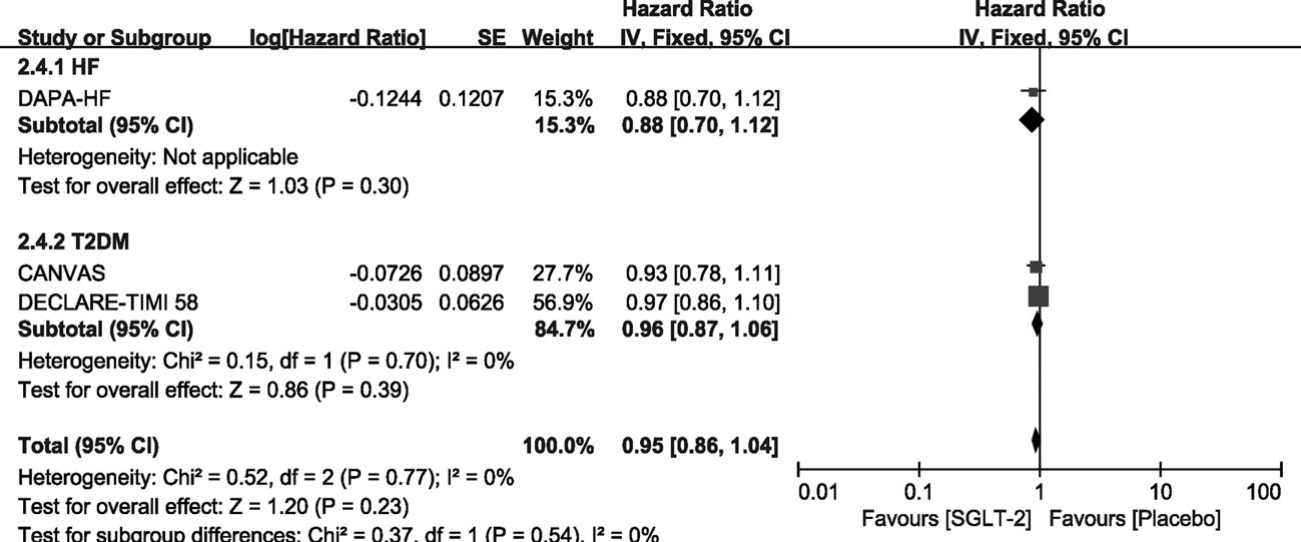
Outcome of all-cause mortality in patients with T2DM or HF.

We carried out exploratory analysis to identify if participants with T2DM/HF will benefit more from the treatment. Except for the SOLOIST-WHF trials, the remaining four trials were included in this process. We found that SGLT-2 inhibitors can improve the outcome of CV death or HHF both in HF [HR, 0.75(95%CI, 0.65-0.87), P = 0.00001] and T2DM [HR, 0.88(95%CI, 0.78-0.99), P = 0.03] groups. It also reduced the incidence of HHF in the HF [HR, 0.67(95%CI, 0.56-0.81), P < 0.0001] and T2DM [HR, 0.77(95%CI, 0.65-0.91), P = 0.002] groups, as shown in **Supplementary File 4**. However, SGLT-2 inhibitors did not lead to an improvement of outcome of CV deaths and all-cause mortality in participants with T2DM or HF alone, as shown in **Figures 6**, **7**.

### Safety Outcome

Safety outcomes of each included trials were presented in different formats, thus, we summarized the major outcomes in **Supplementary File 5**. Overall, SGLT-2 inhibitors were well tolerated in patients with T2DM and HF.

## Discussion

In this systematic review and meta-analysis, we included patients with T2DM and HF who received long-term treatment with SGLT-2 inhibitors. Compared with placebo, the use of SGLT-2 inhibitors is associated with a 31%, 20%, 33%, and 26% reduction in the risk of CV death or HHF, CV death, HHF, and all-cause mortality, respectively. We also conducted exploratory analyses in participants with T2DM/HF. The results showed that although SGLT-2 inhibitors can help reduce the risk of CV death or HHF (27% overall, 25% for HF, 12% for T2DM) and HHF (28% overall, 33% for HF, 23% for T2DM), no significant differences were observed in CV death and all-cause mortality.

It is now known that T2DM and HF represent a risk factor for the other, and many patients should receive combination therapy for diabetes and HF. Recent studies have reported that some anti-diabetes drugs may have beneficial effects in HF, including dipeptidyl peptidase-4 (DPP-4) inhibitors, glucagon-like peptide-1 receptor agonists (GLP-1 RAs), and SGLT-2 inhibitors. Our previous meta-analysis demonstrated that DPP-4 inhibitor and GLP-1 Ras may help improve exercise capacity in HF patients with or without diabetes (29), but these trials showed no improvement in LVEF (30–32), except in a trial with a small sample size (33). SGLT-2 inhibitors may be the most promising choice among these three kinds of drugs detailed above, according to recent meta-analyses (34–37).

Although many studies have investigated the effects of SGLT-2 inhibitors for HF treatment, it was initially developed as an anti-diabetes drug (6) and has not been proven to be useful in HF. Moreover, short-term treatment may lead to inconsistent result compared with long-term treatment. This was demonstrated by the fact that those treated with SGLT-2 inhibitors for 36 weeks and 12 months showed a significantly improvement in NT-proBNP or BNP (13, 23), however, improvement was not observed among patients who underwent 12 and 24 weeks of treatment (12, 21, 22). Thus, we focused on the population with T2DM and HF in this study. The study end points included long-term outcomes of HHF and mortality (either CV death or all-cause mortality). Our study included trials with the shortest median duration of 9 months, and the results showed that both the primary and secondary outcomes were significantly improved. Thus, long-term treatment with SGLT-2 inhibitors in patients with T2DM and HF can reduce the incidence of CV death, HHF, and all-cause mortality.

After completing the study selection process, we found that only the SOLOIST-WHF trial included all participants with T2DM and HF, while data from the remaining four trials were extracted from their subgroup analyses. We wondered if it would lead to any risk of bias; however, in the subgroup analyses reports, we did not find a significant difference in the baseline characteristics. Furthermore, a recent meta-analysis also used data from subgroup analyses (38). Thus, we considered our result credible.

In our study, the included trials recruited participants with different subtype of HF, as well as different status. The CANVAS and SOLOIST-WHF trials included HF patients regardless of EF, while the DAPA-HF, DECLARE-TIMI 58, and EMPEROR-Reduced trials only included HF patients with reduced EF. Furthermore, the CANVAS and SOLOIST-WHF trials included patients with a history of HF, but they did not report if they had existing HF during the randomization period. The DECLARE-TIMI 58 trails classified patients with existing HF and those without existing reduced EF but with HF history, while the other 2 trials only included patients with existing HF. We conducted sensitivity analyses that included patients with reduced EF or existing HF only. The forest plots consisted of our main results, as shown in **Supplementary File 3**. We also took into consideration other variables that may result in incredible conclusions and conducted sensitive analyses, as shown in **Supplementary File 3**. Overall, the sensitivity analyses confirmed the stability of our results.

In this study, we performed an exploratory analysis and tried to identify which population will benefit more from the treatment. Interestingly, although the use of SGLT-2 inhibitors reduced the risk of CV death or HHF, as well as HHF, no changes were observed concerning the reduction of CV death and all-cause mortality in patients with T2DM or HF alone. Thus, our results suggest that patients with T2DM and HF may benefit more from SGLT-2 inhibitors, but further studies are needed to verify this.

This study has several limitations. First, the CANVAS and SOLOIST-WHF trials included HF patients regardless of EF, although we conducted sensitive analyses that only included HF patients with reduced EF, and the results were consistent. We do not know if the results were consistent in HF patients with preserved EF.

Second, in the CANVAS and SOLOIST-WHF trials, all participants had T2DM with a history of HF, but whether they still had HF during the randomization period is unknown. Although we conducted a sensitive analysis, our results could not demonstrate if T2DM patients with HF history but without existing HF would benefit from the treatment.

Third, the CANVAS trial includes two trials, CANVAS including 4,330 participants and CANVAS-Renal including 5,812 participants. In the CANVAS trial, patients were assigned in a 1:1:1 ratio to canagliflozin 300 mg, canagliflozin 100 mg, or the matching placebo. In the CANVAS-Renal trial, patients were assigned in a 1:1 ratio to receive canagliflozin or placebo, but the dose increased from 100 mg in week 1 to 300 mg in week 13. In the SOLOIST-WHF trial, the dose increased from 200 mg to 400 mg, depending on side effects. This may lead to an uncertain risk of bias. However, we think the dose will not have effects on our results.

Finally, these studies recruited participants with different comorbidities, so that they also had a wide range of background medications, which were difficult to summarize. Thus, we could not conduct a sensitive analysis based on the same. However, we believe it will not have an impact on our results.

## Conclusion

Compared with placebo, long-term use of SGLT-2 inhibitors can reduce the risk of CV death or HHF, CV death, HHF, and all-cause mortality in patients with T2DM and HF. This conclusion is most credible in those with existing HF with reduced EF. Moreover, patients with T2DM and HF may benefit more from the treatment.

## Data Availability Statement

The original contributions presented in the study are included in the article/**Supplementary Material**. Further inquiries can be directed to the corresponding author.

## Author Contributions

CC, ML, XL, and GD contributed to the design of the study. CC and HP contributed to acquisition of data. CC, HP, and YZ performed the analyses. CC, ML, XL, and GD contributed to the interpretation of data. CC and GD drafted the manuscript. All authors contributed to critically revise the manuscript. All authors contributed to the article and approved the submitted version.

## Funding

This study was funded by Sanming Project of Medicine in Shenzhen, SZSM201812056.

## Conflict of Interest

The authors declare that the research was conducted in the absence of any commercial or financial relationships that could be construed as a potential conflict of interest.

## Publisher’s Note

All claims expressed in this article are solely those of the authors and do not necessarily represent those of their affiliated organizations, or those of the publisher, the editors and the reviewers. Any product that may be evaluated in this article, or claim that may be made by its manufacturer, is not guaranteed or endorsed by the publisher.
