# Supplementary material for: Patients With Type 2 Diabetes Mellitus and Heart Failure Benefit More From Sodium-Glucose Cotransporter 2 Inhibitor: A Systematic Review and Meta-Analysis
Source: Front Endocrinol (Lausanne). 2021 Oct 25;12:664533. doi: 10.3389/fendo.2021.664533 (PMC8572881; doi:10.3389/fendo.2021.664533)
Supplement: Supplementary file 1 [file DataSheet_1.docx]

**Supplementary 1. Search strategy**


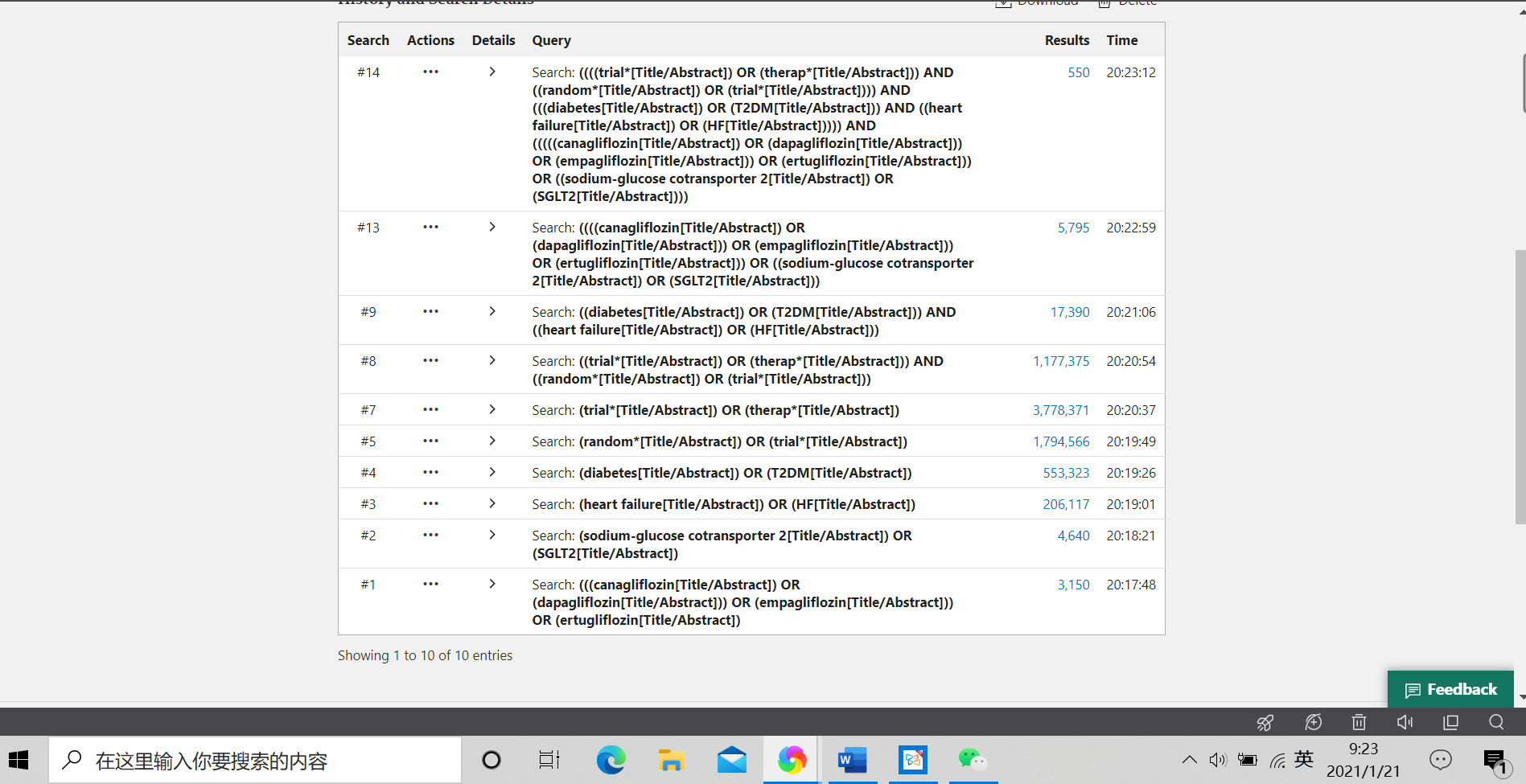


1.1 Search strategy in PubMed, we also recorded the time we performed electronic search


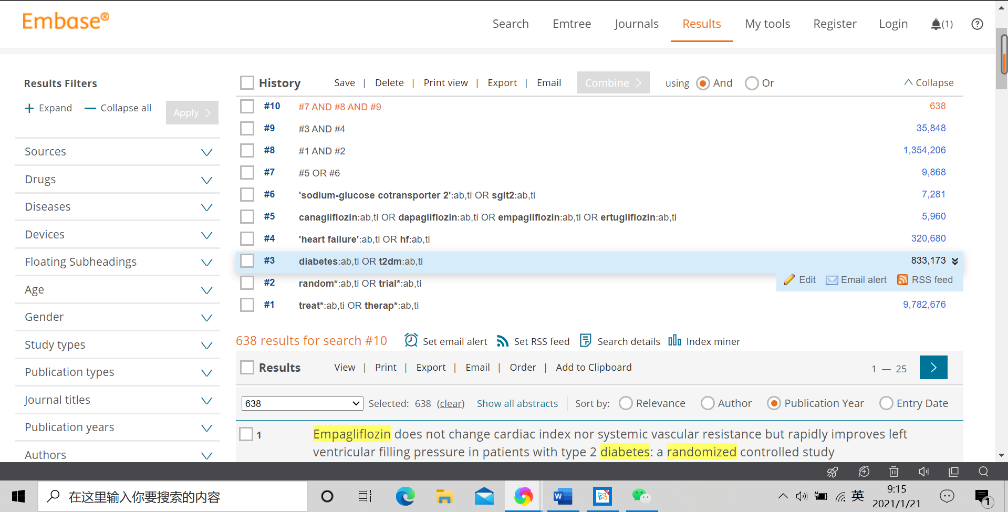


1.2 Search strategy in Embase, we also recorded the time we performed electronic search


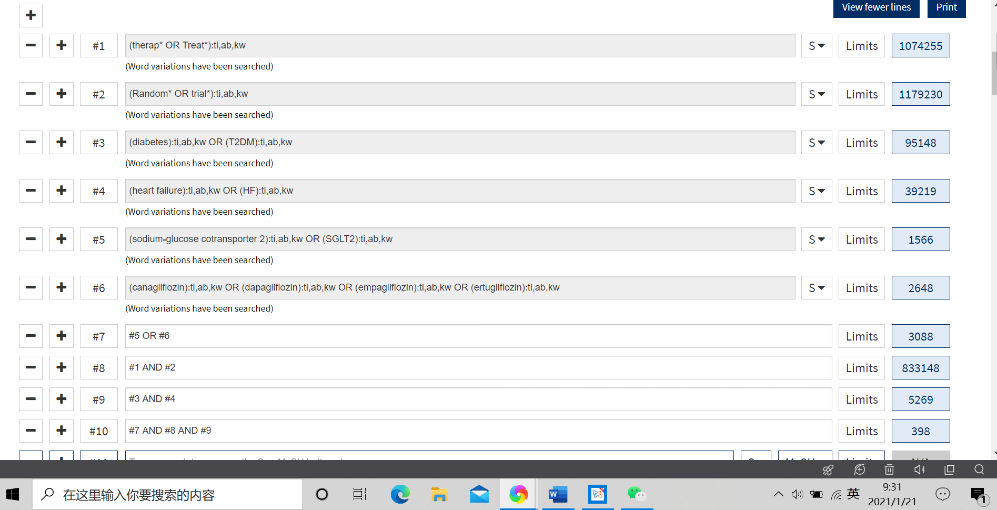


1.3 Search strategy in Cochrane database of clinical trials, we also recorded the time we performed electronic search. The number of records is 398, including one review record.
