# Supplementary material for: Patients With Type 2 Diabetes Mellitus and Heart Failure Benefit More From Sodium-Glucose Cotransporter 2 Inhibitor: A Systematic Review and Meta-Analysis
Source: Front Endocrinol (Lausanne). 2021 Oct 25;12:664533. doi: 10.3389/fendo.2021.664533 (PMC8572881; doi:10.3389/fendo.2021.664533)
Supplement: Supplementary file 2 [file DataSheet_2.docx]

Supplementary 2. Details of risk of bias assessment

| **Trial: DAPA-HF**  **DOI: 10.1056/NEJMoa1911303** | | |
| --- | --- | --- |
| **Bias** | **Authors' judgement** | **Support for judgement** |
| Random sequence generation | Low risk | Assign patient a unique enrolment number, beginning with ‘E#’, which will be used to identify the patient throughout the study. The enrolment code (E-code) will be assigned in the Interactive Voice/Web Response System (IxRS).  The IxRS will allocate the investigational product (IP) through a randomization scheme and provide the randomization number and the appropriate Kit IDs from IP available at the study site. |
| Allocation concealment | Low risk | Randomization will be performed via the Interactive Voice/Web Response System. |
| Blinding of participants and personnel | Low risk | The blinding of treatment is ensured by using a double-blind technique.  No member of the extended AZ study team, personnel at study sites, or any CRO handling study data will have access to the randomization scheme during the study |
| Blinding of outcome assessment | Low risk | Potential endpoints in the study, including the primary efficacy endpoint as well as the secondary and to some extent the exploratory endpoints, will be centrally adjudicated by an independent Clinical Event Adjudication (CEA) committee. |
| Incomplete outcome data | Low risk | A total of 4744 patients were enrolled in this study, and all of these participants were involved in the final analysis. |
| Selective reporting | Low risk | Study protocol available and all pre-specified outcomes are reported.  Patients were evaluated 14 and 60 days after randomization, with additional study visits at 4 months and at 4-month intervals thereafter. The full schedule of assessments is provided in the protocol. |
| Other bias | Low risk | None detected |
| **Trial: CANVAS**  **DOI:**[**10.1056/NEJMoa1611925**](https://doi.org/10.1056/nejmoa1611925) | | |
| **Bias** | **Authors' judgement** | **Support for judgement** |
| Random sequence generation | Low risk | Participants were randomized centrally through an interactive web response system using a computer-generated randomization schedule prepared by the study sponsor using randomly permuted blocks. |
| Allocation concealment | Low risk | Participants were randomized centrally through an interactive web response system. |
| Blinding of participants and personnel | Low risk | This is a randomized, double-blind, placebo-controlled, multicenter study  Participants and all study and sponsor staff were masked to individual treatment allocations until the completion of the study. |
| Blinding of outcome assessment | Low risk | An independent Endpoint Adjudication Committee will assess all events. |
| Incomplete outcome data | Low risk | These two trials involved a total of 10,142 participants, 4330 in CANVAS and 5812 in CANVAS-R. A total of 9734 participants (96.0%) completed the trial. Vital status was confirmed for 10,100 of the 10,142 participants (99.6%).  In the subgroup analysis, all of the 10142 participants were involved in analyses. |
| Selective reporting | Low risk | Study protocol available and all pre-specified outcomes are reported.  After randomization, face-to-face follow-up was scheduled in three visits during the first year and at 6-month intervals thereafter, with telephone follow-up between face-to-face assessments. Every follow-up included inquiry about primary and secondary outcome events and serious adverse events. |
| Other bias | Low risk | None detected |

| **Trial: DECLARE-TIMI 58**  **DOI:  10.1056/NEJMoa1812389** | | |
| --- | --- | --- |
| **Bias** | **Authors' judgement** | **Support for judgement** |
| Random sequence generation | Low risk | Randomization numbers will be prepared by the global randomization administrator at AstraZeneca and made available for IVRS/IWRS use. |
| Allocation concealment | Low risk | Randomization to investigational product will be done via an IVRS/IWRS at Visit 2 via an Interactive Voice/Web Response System (IVRS/IWRS) |
| Blinding of participants and personnel | Low risk | The blinding is ensured by using double-blind, single-dummy technique.  No member of the study delivery team at AZ, BMS, Hadassah, TIMI, or personnel at study centers or any clinical research organization (CRO) handling data will have access to the randomization scheme during the conduct of the study. |
| Blinding of outcome assessment | Low risk | An independent, blinded Clinical Event Adjudication (CEC) will adjudicate all primary and secondary CV endpoints |
| Incomplete outcome data | Low risk | A total of 17,160 participants completed the run-in phase and were eligible to undergo randomization. A total of 3962 patients discontinued the trial regimen prematurely, at a rate of 5.7% per year, including 1811 of 8574 patients (21.1%) in the dapagliflozin group and 2151 of 8569 (25.1%) in the placebo group. Rates of withdrawal of consent (224 patients, at a rate of 0.3% per year) and loss to follow-up (30 patients, at a rate of <0.1% per year) were low and did not differ between the two groups |
| Selective reporting | Low risk | Study protocol available and all primary and secondary outcome variables are reported.  Patients were to return for in-person follow-up every 6 months until trial completion for laboratory testing and assessment of clinical and safety events and adherence to the trial regimen. Patients were contacted by telephone every 3 months between in-person visits. |
| Other bias | Low risk | None detected |

| **Trial: EMPEROR-Reduced**  **DOI: 10.1056/NEJMoa2022190** | | |
| --- | --- | --- |
| **Bias** | **Authors' judgement** | **Support for judgement** |
| Random sequence generation | Unclear risk | Insufficient information in article or rationale and design publication. |
| Allocation concealment | Low risk | Randomization was performed with an interactive-response system that used a permuted-block design |
| Blinding of participants and personnel | Low risk | Patients, Investigators and everyone involved in trial conduct or analysis or with any other interest in this double-blind trial, will remain blinded with regard to the randomised treatment assignments until after database lock. |
| Blinding of outcome assessment | Low risk | An Endpoint Adjudication Committee is evaluating all reported and potential clinical events in a manner blinded to the treatment assignment. An independent Data Monitoring Committee is responsible for ongoing evaluation of the data that accrue during the course of the trial. |
| Incomplete outcome data | Low risk | Four patients in the placebo group did not receive placebo. 303 patients (16.3%) in the empagliflozin group and 335 patients (18.0%) in the placebo group stopped prematurely for reasons other than death. A total of 21 patients (0.6%) had unknown vital status at the end of the trial; 42 patients were lost to follow-up at various times before the data cutoff. |
| Selective reporting | Low risk | Study protocol available. The primary outcome was reported and most of the secondary outcomes were reported.  Every 2 to 3 months, we evaluated patients’ outcomes and adverse events. |
| Other bias | Low risk | None detected |

| **Trial: SOLOIST-WHF**  **DOI: 10.1056/NEJMoa2030183** | | |
| --- | --- | --- |
| **Bias** | **Authors' judgement** | **Support for judgement** |
| Random sequence generation | Low risk | The study biostatistician provides the randomization scheme to the Interactive Response Technology (IRT). Then, the IRT generates the patient randomization list from which it allocates treatment to the patients. |
| Allocation concealment | Low risk | Randomization was performed centrally with the use of interactive-response technology according to the baseline characteristics. |
| Blinding of participants and personnel | Low risk | Double-blind, placebo-controlled, parallel-group design.  To maintain blinding, sotagliflozin and placebo tablets and packaging will be blinded and indistinguishable.  Investigators will not have access to the Randomization (treatment) code. |
| Blinding of outcome assessment | Low risk | An independent Clinical Endpoint Committee (CEC) will review and adjudicate all events of death, selected CV and renal events, diabetic ketoacidosis (DKA), and bone fractures in a treatment-blinded manner. |
| Incomplete outcome data | Low risk | 1222 patients were randomly assigned to a trial group, and all of the patients were included in the efficacy analyses.  At the time of database lock (August 10, 2020), vital status was available for 97.1% of the patients; 43 patients (3.5%) did not complete the final trial visit, among whom vital status was unknown at the end of the trial for 35.  Early discontinuation of the trial regimen for reasons other than death or early termination of the trial occurred in 79 patients (13.0%) in the sotagliflozin group and in 94 patients (15.3%) in the placebo group |
| Selective reporting | Low risk | Study protocol available.  The primary outcome was reported, but there were 2 of 6 secondary outcomes not reported.  Follow-up visits were scheduled at 1, 2, and 4 weeks, at 4 months, and every 4 months thereafter. |
| Other bias | Low risk | None detected |
