# Supplementary material for: Patients With Type 2 Diabetes Mellitus and Heart Failure Benefit More From Sodium-Glucose Cotransporter 2 Inhibitor: A Systematic Review and Meta-Analysis
Source: Front Endocrinol (Lausanne). 2021 Oct 25;12:664533. doi: 10.3389/fendo.2021.664533 (PMC8572881; doi:10.3389/fendo.2021.664533)
Supplement: Supplementary file 3 [file DataSheet_3.docx]

**Supplementary 3. Sensitive analysis**

**3.1 Sensitive analyses of the DECLARE-TIMI 58 trial, we collected the data combined existing HF and with HF history but not existing reduced EF.**


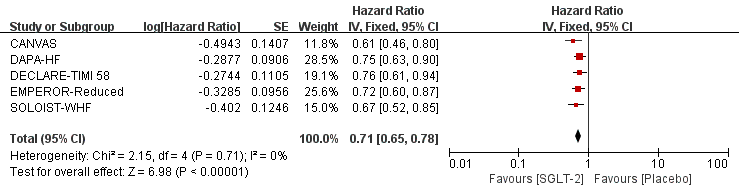


Figure 3.1.1. Forest plot-- DECLARE-TIMI 58 trial-- CV death or HHF


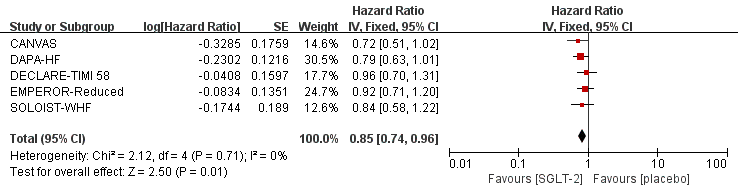


Figure 3.1.2. Forest plot-- DECLARE-TIMI 58 trial-- CV death


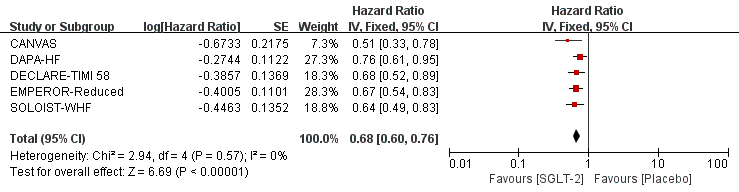


Figure 3.1.3. Forest plot-- DECLARE-TIMI 58 trial-- HHF


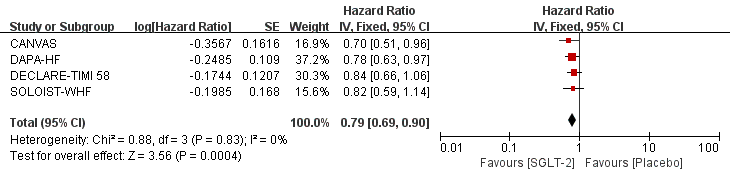


Figure 3.1.4. Forest plot-- DECLARE-TIMI 58 trial-- all caused mortality

**3.2 Sensitive analyses of HHF including first and recurrent HHF, or time to first HHF only**


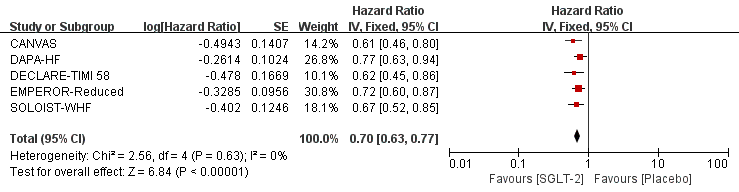


3.2.1 Forest plot-- DAPA-HF-- CV death or HHF (first and recurrent HHF)


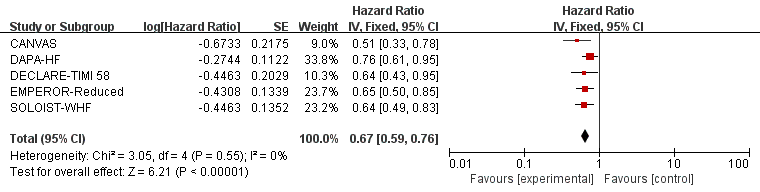


3.2.2 Forest plot-- EMPEROR-Reduced-- first and recurrent HHF

**3.3 Sensitive analyses of different type of HF that only included HF with reduced EF, and different status of HF that only included existing HF. We did not repeat these processes because they had the same data.**


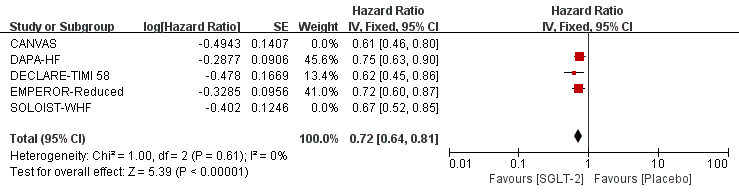


3.3.1 Forest plot--HF with reduced EF/existing HF only--CV death or HHF


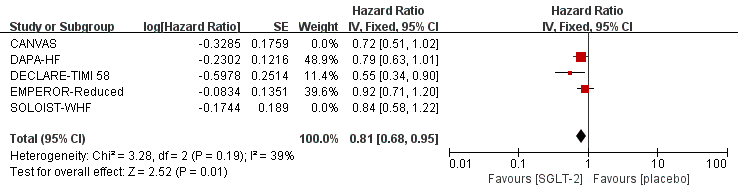


3.3.2 Forest plot--HF with reduced EF/existing HF only--CV death


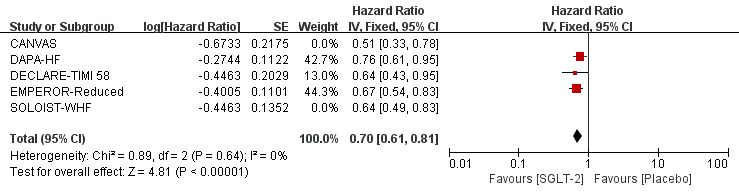


3.3.3 Forest plot--HF with reduced EF/existing HF only--HHF


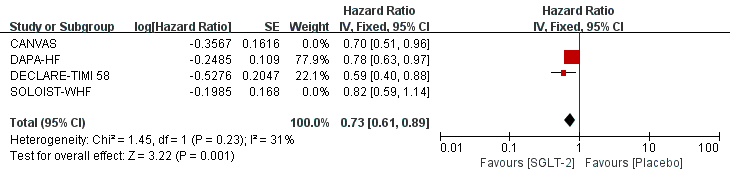


3.3.4 Forest plot--HF with reduced EF/existing HF only—all caused mortality
