# Supplementary material for: Patients With Type 2 Diabetes Mellitus and Heart Failure Benefit More From Sodium-Glucose Cotransporter 2 Inhibitor: A Systematic Review and Meta-Analysis
Source: Front Endocrinol (Lausanne). 2021 Oct 25;12:664533. doi: 10.3389/fendo.2021.664533 (PMC8572881; doi:10.3389/fendo.2021.664533)
Supplement: Supplementary file 4 [file DataSheet_4.docx]

**Supplementary 4**


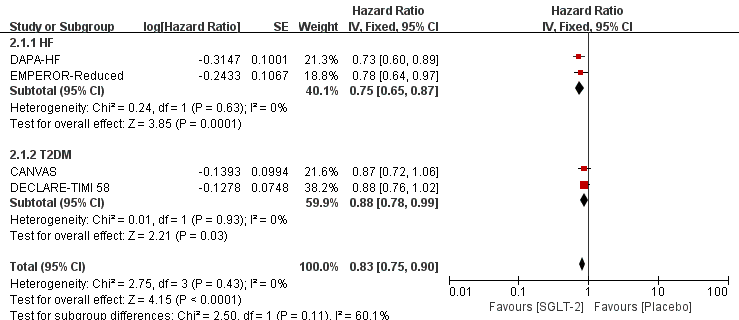


Supplementary 4.1. subgroup analysis of CV death or HHF in participants with T2DM or HF only.


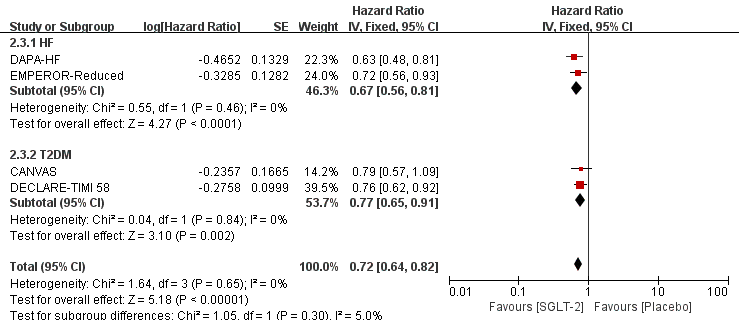


Supplementary 4.2. subgroup analysis of HHF in participants with T2DM or HF only.
