# Supplementary material for: Patients With Type 2 Diabetes Mellitus and Heart Failure Benefit More From Sodium-Glucose Cotransporter 2 Inhibitor: A Systematic Review and Meta-Analysis
Source: Front Endocrinol (Lausanne). 2021 Oct 25;12:664533. doi: 10.3389/fendo.2021.664533 (PMC8572881; doi:10.3389/fendo.2021.664533)
Supplement: Supplementary file 5 [file DataSheet_5.docx]

**Supplementary 5**

Adverse events of the subgroup analyses/main study comparing SGLT-2 inhibitors VS placebo in patients with T2DM and HF

| Study | Intervention | SAE | AE leading to discontinuation | Hypoglycemia | Amputation | Fraction | Volume depletion | Kidney injury events |
| --- | --- | --- | --- | --- | --- | --- | --- | --- |
| CANVAS│ | Canagliflozin | 158.2 | 39.1 | NR | 9.8 | 15.5 | 21.2 | 1.1 |
|  | Placebo | 196.5 | 36.1 | NR | 4.2 | 12.2 | 14.9 | 4.2 |
| DAPA-HF | Dapagliflozin | 447(41.7%) | 43(4.0%) | 4(0.4%) | 12(1.1%) | 22(2.1%) | 84(7.8%) | 91(8.5%) |
|  | Placebo | 513(48.3%) | 57(5.4%) | 4(0.4%) | 9(0.8%) | 25(2.4%) | 83(7.8%) | 92(8.7%) |
| DECLARE–TIMI 58 | Dapagliflozin | 172(56.9%) | NR | 7(2.5%) | 11(3.6%) | 23(7.7%) | 22(7.5%) | 23(8.2%) |
|  | Placebo | 196(58.8%) | NR | 10(3.7%) | 7(2.4%) | 20(6.6%) | 15(5.6%) | 40(14.0%) |
| EMPEROR-Reduced | Empagliflozin | 397(42.8%) | 175(18.9%) | 20(2.2%) | 12(1.3%) | 20(2.2%) | 103(11.1%) | 98(10.6%) |
|  | Placebo | 457(49.4%) | 176(19.0%) | 22(2.4%) | 9(1.0%) | 26(2.8%) | 84(9.1%) | 98(10.6%) |
| SOLOIST-WHF | Sotagliflozin | 235(38.8%) | 29(4.8%) | 26(4.3%) | 4(0.7%) | 12(2.0%) | 57(9.4%) | 25(4.1%) |
|  | Placebo | 251(41.1%) | 23(3.8%) | 17(2.8%) | 1(0.2) | 9(1.5%) | 54(8.8%) | 27(4.4%) |

T2DM: type 2 diabetes mellitus; HF: heart failure; SAE: serious adverse events; AE: adverse events; NR: not reported

Data were presented with n (%), except the CANVAS study was presented with patients per 1000 patients-years
